# Genomic analysis of the regulatory elements and links with intrinsic DNA structural properties in the shrunken genome of *Buchnera*

**DOI:** 10.1186/1471-2164-14-73

**Published:** 2013-02-01

**Authors:** Lilia Brinza, Federica Calevro, Hubert Charles

**Affiliations:** 1 UMR203 BF2I, Biologie Fonctionnelle Insectes et Interactions, INSA-Lyon, INRA, Université de Lyon, Villeurbanne, France; 2 BAMBOO, INRIA Rhône-Alpes, Montbonnot Saint-Martin, France; 3 Present address: Université de Lyon, Université Lyon1, CNRS, UMR 5558, Laboratoire de Biométrie et Biologie Evolutive, Lyon, France

**Keywords:** *Buchnera aphidicola*, Genome reduction, Transcription regulation, DNA-topology, Nucleoid associated proteins (NAPs)

## Abstract

**Background:**

*Buchnera aphidicola* is an obligate symbiotic bacterium, associated with most of the aphididae, whose genome has drastically shrunk during intracellular evolution. Gene regulation in *Buchnera* has been a matter of controversy in recent years as the combination of genomic information with the experimental results has been contradictory, refuting or arguing in favour of a functional and responsive transcription regulation in *Buchnera*.

The goal of this study was to describe the gene transcription regulation capabilities of *Buchnera* based on the inventory of cis- and trans-regulators encoded in the genomes of five strains from different aphids (*Acyrthosiphon pisum*, *Schizaphis graminum*, *Baizongia pistacea*, *Cinara cedri* and *Cinara tujafilina*), as well as on the characterisation of some intrinsic structural properties of the DNA molecule in these bacteria.

**Results:**

Interaction graph analysis shows that gene neighbourhoods are conserved between *E*. *coli* and *Buchnera* in structures called transcriptons, interactons and metabolons, indicating that selective pressures have acted on the evolution of transcriptional, protein-protein interaction and metabolic networks in *Buchnera*. The transcriptional regulatory network in *Buchnera* is composed of a few general DNA-topological regulators (Nucleoid Associated Proteins and topoisomerases), with the quasi-absence of any specific ones (except for multifunctional enzymes with a known gene expression regulatory role in *Escherichia coli*, such as AlaS, PepA and BolA, and the uncharacterized hypothetical regulators YchA and YrbA). The relative positioning of regulatory genes along the chromosome of *Buchnera* seems to have conserved its ancestral state, despite the genome erosion. Sigma-70 promoters with canonical thermodynamic sequence profiles were detected upstream of about 94% of the CDS of *Buchnera* in the different aphids. Based on Stress-Induced Duplex Destabilization (SIDD) measurements, unstable σ^70^ promoters were found specifically associated with the regulator and transporter genes.

**Conclusions:**

This genomic analysis provides supporting evidence of a selection of functional regulatory structures and it has enabled us to propose hypotheses concerning possible links between these regulatory elements and the DNA-topology (i.e., supercoiling, curvature, flexibility and base-pair stability) in the regulation of gene expression in the shrunken genome of *Buchnera*.

## Background

*Buchnera aphidicola*, associated with most of the aphids (Hemiptera: aphididae), is a fascinating bacterium, both because of its apparent minimalist physiology and because of its intermediate status between an autonomous cell and an intracellular organelle. Shaped by some 150–200 million years of intracellular evolution, its genome and regulatory system have evolved to fit the evolutionary constraints imposed by the symbiotic partnership [1,2]. This work is a comparative genomic analysis of cis- and trans-regulators encoded in the *Buchnera* genomes of five different aphid strains, *Acyrthosiphon pisum* (*BAp*), *Schizaphis graminum* (*BSg*), *Baizongia pistacea* (*BBp*), *Cinara cedri* (*BCc*) and *Cinara tujafilina* (*BCt*), combined with analyses of the intrinsic physical topological properties of their DNA molecules. The main objective was to decipher the regulatory mechanisms underlying gene regulation in these bacteria.

The *Buchnera* genomes from the five aphid species share certain properties: (1) a small size, from 416 kb for *BCc* to 641 kb for *BAp*[1,3-6]; (2) a low GC-content of about 25%; (3) a standard bacterial gene density of about 85% of coding DNA; (4) the conservation of most genes encoding enzymes from the biosynthesis of essential amino acids that *Buchnera* furnish to their hosts [7,8]. The differences between these five aphid species are related to the physiology of the symbiotic interactions that created specific evolutionary constraints, contributing to the differentiation of the *Buchnera* gene repertoires [9]. *BCc* offers an example of evolution with an extremely reduced genome, probably linked to the presence in their aphid host of the co-primary endosymbiont “*Candidatus* Serratia symbiotica” with which they show a strong dependency and partially share genes of several amino acid biosynthetic pathways [10,11].

Gene regulation in *Buchnera* has been a matter of controversy in recent years. Global transcriptomic analyses revealed a weak transcriptional response to various stresses applied on the host, such as heat shock [12] and single amino acid excess [13] in *BSg*, as well as aromatic and essential amino acid depletions in *BAp*[14]. However, stronger effects were observed when the transcriptional responses were compared between *Buchnera* populations from embryonic and maternal aphid compartments [15], somehow reflecting two different physiological growing states of *Buchnera*. Finally, following the kinetics of the response in *BAp*, a specific induction (repression) of the genes of the leucine biosynthetic pathway was observed after one day of treatment following a depletion (excess) of the leucine concentration in the aphid diet, although the transcriptional response was not significant after seven days of treatment [16].

In bacteria, two main interrelated processes govern gene transcription [17]. There is the “classical” mechanism, involving sigma and specific transcription factors binding DNA sequences located in the proximity of the transcrip-tion initiation site of a gene and able to induce or repress transcription initiation by the RNA polymerase [18]. Then there is a more recently discovered mechanism based on the regulation of DNA topology controlled by Nucleoid Associated Proteins (NAP) and topoisomerases [19-23] and partially characterized by several physical parameters, such as DNA stability, curvature and supercoiling [24,25]. Both processes involve trans-regulatory factors (*i*.*e*., proteins binding to DNA) interacting, with varying degrees of specificity, with the cis-regulatory elements (*i*.*e*., DNA sequences). The regulatory mechanisms controlling transcription initiation are the most thoroughly described in free-living bacteria [18] and genome organisation suggests that they have also been conserved in *Buchnera*[26]. Other events responsible for transcriptional regulation, like termination, mRNA maturation and stability control, as well as translation regulation, are also important targets for gene expression regulation, possibly involving small RNAs [27,28]. These mechanisms have not been taken into account in this work. Finally, post-translational modification by reversible N^ε^-lysine acetylation of transcription factors has been recently reported in bacteria and might directly affect gene expression [29]. However, it seems unlikely that this mechanism exists in *Buchnera* as the corresponding acetyltransferase (Pat or YfiQ) and the NAD-dependant deacetylase (CobB), described in *Salmonella enterica* and *Escherichia coli*, are lacking in *Buchnera*.

The bacterial chromosome is known to be associated with proteins which allow for a massive compaction and, at the same time, are able to dynamically regulate the DNA molecule, rendering rapidly accessible those DNA regions which need to be transcribed [30,31]. NAPs have been extensively described in *E*. *coli*[32,33]. They participate in the chromosome structuring and also in all the processes involving DNA transactions (replication, recombination and transcription). NAPs are basic, small molecular weight proteins and their relative abundance is dynamic and dependent on the cell physiology. For example, different bacterial growing phases are characterised by specific expression patterns of the different NAPs [34,35]. Although more than 12 NAPs have been described in *E*. *coli*, almost all the literature centres on only four of them: H-NS (Histone-like Nucleoid Structuring protein), HU (Heat Unstable nucleoid protein), IHF (Integration Host Factor) and FIS (Factor for Inversion Stimulation). Apart from NAPs, the maintenance of the chromosome supercoiling in bacteria is controlled by topoisomerases that either relax the negative supercoils (type I topoisomerase) or serve to introduce them (ATP consuming type II topoisomerase), hence linking the energetic metabolism of the cell with the DNA topology [36].

Negative supercoiling is essential for chromosome compaction and for the survival of the bacteria [21,37]. Local supercoiling variations in the DNA modulate the polymerase affinity for promoters. Hence, DNA supercoiling is often considered as a true transcriptional regulator that is sensitive to the environmental conditions [19,23,35,38] and that uses the ATP/ADP ratio governing gyrase activity as a sensor of the energetic level of the cell [36,39]. Curvature, flexibility and stability, contrary to supercoiling, are properties which are highly correlated with the primary sequence of the DNA molecule, although it has been suggested that nucleoid proteins might also influence these parameters [40]. Curvature and flexibility (estimated in this work by the base-pair propeller twisting) are essential for the initiation of transcription since the promoter affinity for polymerase, and for the associated transcription factors, is sensitive to the topology of the DNA molecule [24]. The double-strand stability of the DNA molecule is also very important, particularly in the promoter region, for transcription initiation. In this study, we have estimated the base stacking energy, which is a direct measure of the base pair affinity within the DNA molecule [41], and the Stress-Induced DNA Duplex Destabilization (SIDD) [42] to assess the stability of the double strand DNA molecule in *Buchnera*.

The aim of this work is to give an initial description of the structure and of the evolution of the gene regulatory network in *Buchnera* using a genomic comparative analysis. Regulatory networks are known to evolve quickly and both the DNA-binding domains of transcription factors and their target sequence sets are highly dynamic (i.e., orthologous regulators are often regulating non-orthologous targets), making comparative studies difficult [43,44]. The *Buchnera* model is interesting in this respect, first because the bacteria evolved for millions of years sequestrated within aphids, preventing any contact with other bacterial populations and, hence, any horizontal gene transfer and, secondly, because *Buchnera* were almost uniformly constrained by the intracellular conditions (which relaxes the selection of some genes that become superfluous) and by the physiological requirements imposed by their symbiotic association with aphids (mostly concerning the biosynthesis of essential metabolites, such as amino acids). Thus, after analysing the selective constraints exerted on the regulatory genes, we performed a systematic characterization of the cis- and trans-regulatory elements predicted in the *Buchnera* genomes from the five sequenced strains. These analyses were then coupled with the characterisation of some intrinsic topological properties of the *Buchnera* DNA chromosome, which allowed us to formulate certain hypotheses regarding their possible involvement in gene transcription regulation in these bacteria.

## Methods

### *Buchnera* strains and GenBank sequence accession numbers

Four *B*. *aphidicola* genomes (*BAp*, *BSg*, *BBp*, *BCc*) from the following four aphids: *Acyrthosiphon pisum*, BA000003 [1]; *Schizaphis graminum*, AE013218 [3]; *Baizongia pistaciae*, AE016826 [4]; *Cinara cedri*, CP000263 [5], were used in this work. A fifth genome sequence was published recently from the aphid *Cinara tujafilina* (CP001817 [6]), very closely related to *BCc*. As *BCt* and *BCc* show very similar genomic repertoires and properties, *BCt* was not always included in the analyses of this study. The annotations of transcription units (operons) in *BAp* are those described in [26].

In this work, the *E*. *coli* genome was used as the reference ancestral state of the *Buchnera* lineage, disregarding the evolution in the branch of *E*. *coli*. The analyses were performed on the genomic sequence of *Escherichia coli* str. K-12 substr. MG1655 (U00096) [45,46] and information concerning the gene regulatory network was taken from the RegulonDB 6.2 database [47].

### Inventory of the transcription factors encoded in *Buchnera* genomes

The inventory of the transcription factors encoded in the *Buchnera* genomes was primarily carried out using the close proximity of *Buchnera* to *E*. *coli* and the one-to-one orthology relationship for almost all of the genes of *Buchnera*, as referred to in BuchneraBase [48].

We completed this inventory by searching in the *Buchnera* protein set for all Pfam domains annotated as regions with putative regulatory functions [49]. As helix-turn-helix (HTH) domains are by far the most common protein DNA-binding domains in bacteria [50,51], a systematic search for these structural domains was also carried out for the complete set of proteins in *Buchnera*, using the HTH software [52,53]. Non-HTH motif predictions such as zinc fingers, helix-loop-helix, beta-sheet antiparallel or RNA binding domains are available in the generalist DNA Binding Domain Database [54]. Indeed, three non-HTH predictions are available for *Buchnera*: CspE and CspC each with a cold-shock domain and DksA with a zinc finger domain. These proteins were already detected by their orthologous annotations in *E*. *coli*.

### Annotation of the cis-regulatory elements in *Buchnera*

Promoters and transcription starts were predicted, in the four *Buchnera* strains, using the software BPROM^©^ (http://linux1.softberry.com/berry.phtml) and MacVector (MacVector, Cary, NC, USA) for σ^70^ and σ^32^ promoters, respectively. BPROM was calibrated for *E*. *coli* with a specificity of about 80%. In this work, predictions were made using the *E*. *coli* parameters in the 500 bp regions located upstream from all the *Buchnera* coding DNA sequences (CDS). The motif detection function of MacVector was calibrated using the two consensus σ^32^-boxes (CTTGAAAA and CCCCTNT), separated by 11–15 bp [55], with a tolerance of 50% similarity, according to previous work performed in *BSg*[12].

The transcription factor binding sites (TFBS), known in *E*. *coli* for the different NAPs, were searched for in the *Buchnera* genomic sequences using the *words*.*pos* and *gregexpr* R-functions from the *SeqinR*[56] and *base* libraries, respectively. The following consensus motifs were searched: GNTYAWWWWWTRANC for FIS [57], WAT CAANNNNTTR for IHF [58] and TCGWTWAAWW for H-NS [59].

### Statistical analysis and gene ontology annotations

All the statistical analyses were performed using the R software (http://www.r-project.org). The Gene Ontology (GO) annotations were extracted from the UniprotKB-GOA database [60]. Annotations were compared at the same level (i.e., level 3 or 4) to avoid redundancy-bias linked to the possible non-homogenous depth of the different branches of the GO hierarchy.

### Interaction-graph analysis and genome rearrangements

The C3P software, developed by Boyer *et al*. [61], is a graph-theoretical approach extracting common connected components between two or more graphs for exploring gene neighbourhoods in a genomic and functional context. If we consider two genes, x and y, located in close proximity on the genome, the main principle of this method is that if x and y are co-regulated (i.e., neighbours in the transcription regulatory network), or if the proteins X and Y are interacting directly (i.e., neighbours in the protein-protein interaction network), or are catalyzing successive steps in a metabolic pathway (i.e., neighbours in the metabolic network), the relative positioning of the genes x and y should be preserved during genome evolution if selective pressures are acting on the transcriptional, protein-protein interaction or metabolic networks, respectively. In the present study, we have applied this approach to extract the common connected components between the *Buchnera* genome, on the one hand, and the transcription network, protein interaction network and metabolic network on the other hand. Two genes are considered as neighbours if they are separated by a maximum of one gene in the molecular interaction networks and by five genes on the genome. A set of neighbour genes (or of proteins encoded by a set of neighbour genes) is called: (1) a synton in the genome; (2) a transcripton (this term was not defined in the original work of Boyer *et al*. [61]) in the transcriptional regulatory network; (3) an interacton in the protein-protein interaction network; or (4) a metabolon in the metabolic network. The transcriptional network, together with the protein-protein interaction and the metabolic networks in *Buchnera*, were inferred from *E*. *coli* by direct orthology. In order to test the significance of the structures described in *BAp* (i.e., to establish whether they could have been observed by chance), we developed a procedure to simulate random transcriptons (r-transcriptons). For that, X genes were randomly selected, within a limited span of 5 genes in the *E*. *coli* genome, to generate an r-transcripton. The size (X) of each r-transcripton was defined by sampling the sizes of the true transcriptons of *E*. *coli*. The r-transcriptons were retained only if they shared at least two orthologous genes with *BAp*. Each simulation was ended either when 38 r-transcriptons, sharing at least two orthologues with *BAp*, were generated or when all the genes of *E*. *coli* had been used since gene sampling was performed without replacement. Since a number of r-transcriptons equal to, or greater than 37 were found in only 9 out of the 1000 simulations, a p-value of 9. 10^-3^ was estimated (see Results and discussion section and Table 1 for the justification of the thresholds).

**Table 1:** **Fate, in *BAp, *of “ancestral” orthologous interactons, regulons and metabolons found in *E. coli***

	**# of *E. coli *CCC**^**a**^	**# of *E. coli *CCC with 2 genes conserved (at least) in *BAp***	**# of *E. coli *CCC conserved as CCC in *BAp***	**# of *BAp *CCC not found in *E. coli***
Transcriptons	239	38	37	12
Interactons	39	10	9	0
Metabolons	107	23	23	0

^a^: Number (#) of Common Connected Components.

We also analysed the evolutionary history of each transcription unit (TU) making up the *E*. *coli* transcriptons and conserved in *BAp*. For that, several types of TUs were defined: identical TUs are *BAp* TUs with exact orthologous replicates in *E*. *coli* whereas similar TUs are *BAp* TUs that underwent gene deletions in *BAp* (or more rarely gene insertions in *E*. *coli*). Split, merged and reorganized TUs are *BAp* TUs that were recomposed from different “ancestral” TUs during *Buchnera* evolution. More details and schemes are given in [26].

### Physical properties of the DNA molecule of *Buchnera*

Four physical sequence-dependent properties of the DNA molecule were computed using the GeneWiz software [25] for the four sequenced *Buchnera* strains: stress-induced duplex destabilization - SIDD [42], curvature [62], base stacking energy [41] and base-pair propeller-twisting [63]. The calculation of these different parameters with GeneWiz leads to a single value for each base of the DNA sequence. Non-overlapping sliding windows of different sizes were then defined to calculate the smooth parameter-distributions at different scales (e.g., 300 bp for the whole genome sequence analysis and 20 bp for the promoter region analysis).

For the whole genome comparison between *Buchnera* and *E*. *coli*, two null models of base composition were constructed. The first one (global null model) is a uniform permutation of all the bases of the genome (i.e., preserving only the global GC-content on the genome scale) whilst the second one (local null model) is a uniform base permutation applied to each coding and non coding region (i.e. preserving the local GC-content, as well as the coding versus non-coding GC content).

## Results and discussion

### Selective constraints acting on the regulatory structures and genome functional analysis in *Buchnera*

In order to determine whether protein interactions (across protein, metabolic or transcription regulatory networks) exerted a selective pressure on the conservation of some genomic regions in *BAp*, we used the C3P program [61] to calculate the number of interactons, metabolons and transcriptons conserved between *E*. *coli* and *BAp* (see Methods section for the definitions). Essentially the idea is that if selective constraints have an effect on the three latter structures, and if the genes are close neighbours on the *E*. *coli* genome (supposed, here, to reflect the ancestral state), we expect to see conservation of the corresponding orthologous genes in the equivalent neighbourhood in *BAp*. Indeed, Table 1 shows that 37/38, 9/10 and 23/23 of the *E*. *coli* transcriptons, interactons and metabolons, respectively, found in *BAp* are conserved as common connected components (so the proximity between genes was conserved between *E*. *coli* and *Buchnera* for these particular genes). These results are significant (*i.e*., not observed by chance), as revealed by a re-sampling test giving a p-value of 0.009 for the *BAp* transcriptons (see Methods section). The procedure was not applied to the metabolons and interactons because they are bigger structures, with a lower probability of being observed randomly as conserved associated structures.

Moreover, we have shown that, in addition to the 37 “ancestral” transcriptons, 12 supplementary ones have been produced in *BAp*, by genomic rearrangements, since its divergence with *E*. *coli*. We also analysed the evolutionary history of the TUs composing the transcriptons, found in *BAp*, following on from our previous work on TUs in *Buchnera*[26]. Among the 55 TUs composing the 38 transcriptons found in both *BAp* and *E*. *coli*, 16 are monocistronic, 13 are identical or similar TUs (totally or partially conserved from gene deletions) and 26 are TUs that were reorganised during genome evolution, i.e., formed from a fusion or split from different ancestral TUs (see Methods section for definition of the different TU rearrangements). These results reveal that these two bacterial lineages conserved not only some single operonic structures but also some bigger synthenic fragments associating several TUs. Moreover, genomic rearrangements occurring in the *BAp* lineage during genome shrinkage seem to have clustered some co-regulated genes within the same neighbourhood (i.e., new transcriptons).

During the process of genome shrinkage in the *Buchnera* lineages, which followed the symbiotic association with aphids, gene losses occurred preferentially within functional classes that escaped from selective pressure due to the new intracellular environment. A systematic search for under- and over-represented functional classes of genes was performed in *BAp*, relative to those found in *E*. *coli* (Additional file 1A), with a specific focus on gene expression regulation. Hence, as previously mentioned in the primary annotation [1], gene proportions within the GO classes of *transporter activity*, *developmental processes*, *response to stimulus*, *localization* and *biological regulation* are significantly lower in *Buchnera*, compared to *E*. *coli*, whereas proportions of many classes corresponding to central core metabolism (e.g. *catalytic activity*, *metabolic processes* and *cellular processes*) are significantly higher.

Moreover, we demonstrate here that the genes preferentially conserved in *BAp* are mostly the multifunctional ones. Indeed, conserved genes in *BAp* have a significantly higher association with multiple GO annotations since the distribution of the number of GO terms associated with *BAp* genes shows more genes with 2 to 8 GO terms, when compared to *E*. *coli* (Additional file 1B, Wilcoxon rank test p-value = 2 10^-16^). These multifunctional genes are mostly metabolic genes (i.e., associated with the GO term GO:0008152). Indeed, when the analysis is repeated, after removing those genes associated with the corresponding GO term, the distributions remain almost equivalent (Additional file 1C, Wilcoxon rank test p-value = 0.07). This result is important in the context of genome shrinking, demonstrating that genes encoding multifunctional proteins may be favoured in small genomes.

*Buchnera* have lost a number of genes from the catabolic pathways since most of the end products, synthesized by *Buchnera*, are exported to the host (only 9% of the *E*. *coli* catabolic pathways are present in *BAp*). Salvage pathways have been interwoven between the two symbiotic partners during symbiotic evolution [8,64]. It is important to note that catabolic genes are generally more highly regulated in *E*. *coli*, as compared to anabolic genes [65]. Indeed, 83% of the catabolic genes are regulated in *E*. *coli*, versus only 40% for the anabolic genes. Hence, the loss of catabolic genes partly explains the decay of the gene expression regulatory system in *BAp*.

### Inventory of the trans-regulatory elements in *Buchnera* genomes

A systematic search was performed for the complete set of proteins of the four *Buchnera* strains (*BAp*, *BSg*, *BBp* and *BCc*) using one-to-one orthology with annotated genes of *E*. *coli*, scanning for annotated Pfam domains and HTH domains (see Methods section). Nineteen proteins were detected and are presented in Table 2. The corresponding orthologous genes were searched for within the newly sequenced *BCt* genome and are presented in the same table. Although no new regulator was discovered, this is the first time that such a global and comparative analysis across the *Buchnera* strains has been published.

**Table 2:** **Inventory of regulatory proteins detected in *Buchnera***

**Gene name**	**Regulator type**	**Identity**^a^	***BAp***	***BSg***	***BBp***	***BCc***	***BCt***^b^	**Prot**^c^
*rpoD*	σ factors	82.11	+	+	+	+	+	+ (236)
*rpoH*		72.18	+	+	+	+	+	−
*alaS*	“Specific”	50.11	+	+	+	+	+	+ (137)
*bolA*		27.62	+	+	+	+	+	−
*pepA*		52.09	+	+	+	−	+	+ (301)
*metR*		−	pseudo	+	−	−	−	−
*dksA*	Bifunctional	70.89	+	+	+	−	+	+ (218)
*cspC*		91.30	+	−	−	−	−	+ (229)
*cspE*		94.20	+	+	+	+	+	+ (243)
*csrA*		86.89	+	+	+	+	+	−
*ychA*	Hypothetical	57.62	+	+	+	+	+	+ (215)
*yrbA*		41.67	+	+	+	+	+	+ (348)
*fis*	NAPs and topoisomerases	65.31	+	+	−	+	−	+ (366)
*hns*		60.58	+	−	−	−	−	−
*hupA*		66.30	+	+	+	−	−	+ (307)
*himA*		59.80	+	+	−	+	+	+ (321)
*himD*		68.08	+	+	−	+	+	+ (397)
*ybaB*		81.65	+	+	+	+	+	+ (283)
*topA*		55.43	+	+	−	−	−	−
*gyrA*		63.58	+	+	+	+	+	+ (102)
*gyrB*		62.11	+	+	+	+	+	+ (124)

^a^: protein sequence identity (%) with the *E. coli* orthologous gene; ^b^: genome sequence of *BCt* was not analysed systematically, we have only reported here the presence/absence of orthologous genes; ^c^: proteomic data were compiled from [66], +/− (rank) indicates the presence/absence of the protein in partially purified samples of *BAp* (rank of the quantitative detection out of 400 proteins).

#### Sigma factors

Among the six sigma factors that were predicted in the last common ancestor of *E*. *coli* and *Buchnera*[67,68], only two have been conserved in the five *Buchnera* strains analysed in this work: σ^70^, encoded by the *rpoD* gene, which is the constitutive bacterial sigma factor, and σ^32^, encoded by the *rpoH* gene, which is the factor responsible for heat shock regulon transcriptional control. These two proteins show a high sequence identity with those of *E*. *coli*, and the RpoD protein was detected by proteomics analysis in a partially purified *BAp* sample, whereas RpoH (probably repressed in non-stressed conditions) was not ([66] in Table 2). Hence, the σ^24^, σ^28^, σ^38^ and σ^54^ regulons have all been lost during the process of genome reduction in the *Buchnera* lineage, and this loss was probably a long time ago as it occurred before the divergence that gave rise to the evolution of the five *Buchnera* strains analysed here.

#### Transcription factors

Four transcription factors are described as “specific” in our work as they are associated with one, or only a very few, target genes in *E*. *coli*. Three of them (AlaS, PepA and BolA) are present in the genomes of the all *Buchnera* strains we analysed, whereas most of their known putative targets in *E*. *coli* are not conserved. Only AlaS and PepA were detected by proteomics analyses performed in the *BAp* strain [66]. AlaS, encoding alanyl-tRNA synthetase, acts as an autorepressor of the *alaS* gene, sensitive to the concentration of alanine in bacterial cells [69]. In *E*. *coli*, the gene *pepA* encodes the multifunctional DNA-binding enzyme PepA, which is an aminopeptidase also acting as a transcription factor regulating the *carAB* operon and, thus, it is involved in the metabolism of arginine and proline. The fixation domain to the DNA of PepA has been widely studied as it is atypical and does not show classical DNA-binding motifs [70].

A similar example of the conservation, in *Buchnera*, of such multifunctional enzymes with transcriptional regulatory properties is provided by the reductase BolA known in *E*. *coli* for forming iron-sulfur (FeS) clusters with glutaredoxin [71,72]. The expression of BolA was originally described as being exclusively associated with the stationary phase in *E*. *coli*, but BolA is also implicated in the response to a broad range of stress conditions (heat, osmotic, oxidative, acidic and nutritional stresses) [73]. In addition, this protein is described as a morphogene, containing a putative HTH-domain potentially involved in the regulation of genes responsible for the cellular morphology changes in stress conditions [74-76]. Until now, BolA has been shown to be able to regulate only four genes, at a transcriptional level, in *E*. *coli*: the *dacA*, *dacC* and *ampC* genes (involved in penicillin resistance) and the *mreB* gene (involved in rod-shape maintenance) [71,72]. None of these genes have been conserved in the *Buchnera* genomes. Moreover, in *Buchnera*, the HTH DNA-binding domain was not detected with a statistically significant score. Several studies have recently highlighted the transcriptional regulatory role of enzymes in bacteria [77-80]. In *Buchnera*, the absence of two-component sensors might be compensated for by such multifunctional enzymatic systems that combine catalytic and regulatory properties and are directly sensitive to substrate availability. This hypothesis is consistent with the observation that *Buchnera* genomes have selected for the conservation of multifunctional genes (Additional file 1) but it is not, as yet, known whether AlaS, PepA and BolA have acquired a broad regulatory role and a large spectrum of targets, nor whether other enzymes might have been recruited for a similar regulatory function in *Buchnera*.

Finally, the regulator *metR*, involved in the transcriptional regulation of the methionine biosynthetic enzymes, is conserved only in *BSg* whereas it is present as a pseudogene in *BAp* and absent in *BBp*, *BCc* and *BCt*. Moran *et al*. [13] have proposed an evolutionary scenario hypothesizing that the conservation of methionine regulation in *BSg* could be linked to the high variability of the cysteine concentration, supplied as homocysteine, in the *BSg* diet, compared to the other *Buchnera* strains.

Four transcription factors are referred to here as “bifunctional” (CspC, CspE, CsrA and DksA) because of their putative capability to bind both DNA and RNA molecules, although they do not function as classical transcription factors in *E*. *coli*. CspC and CspE are members of the cold-shock protein family of *E*. *coli*, which are also RNA chaperones thought to facilitate translation at low temperatures by destabilizing the mRNA secondary structures. Bae *et al*. [81] have revealed that these proteins also act as transcription anti-terminators, interacting with the Rho-independent termination mechanism. In addition, these proteins are known to play a role in the regulation of DNA-topology, stabilising chromosome compaction and, hence, regulating gene expression [82]. CspC is found only in *BAp*, whereas CspE is conserved in the genomes of the five sequenced *Buchnera* strains. CsrA, conserved in the five *Buchnera* strains, has been identified in *E*. *coli* as a post-transcriptional regulator able to bind to the 5’ untranslated leaders of some mRNAs by competing with the interaction of the 30S ribosomal subunits, thus inhibiting their translation [83]. CsrA may also be involved in indirect gene expression activation by an, as yet, unknown mechanism [84,85]. In *E*. *coli*, CsrA is involved in many cellular processes but it was originally discovered as a global regulator of carbon source metabolism. It is a general activator of glycolysis (which *Buchnera* can do) and a repressor of glyconeogenesis (which *Buchnera* cannot do). CsrA is antagonized, in *E*. *coli*, by the sRNAs CsrB and CsrC that seem to be absent in *Buchnera*. Edwards *et al*. [83] have recently proposed a more general regulatory role for CsrA, describing 721 putative target genes in *E*. *coli*, and they have revealed a link with the stringent response involving the DksA protein. DksA is known, in *E*. *coli*, for its ability to regulate transcription elongation. The protein is able to bind with the RNA polymerase secondary channel, to interact with the alarmone ppGpp, and to destabilize the transcription complexes bound at the discriminator sites of the promoters, hence allowing elongation to start [86-89]. Although the ppGpp alarmone can no longer be produced in *Buchnera* (due to the absence of *relA* and *spoT* genes), DksA could have conserved its regulatory roles in this bacterium. We hypothesize that DksA might destabilize the σ 1.2 sub-region of the σ^70^ factor associated with the AT-rich discriminators of most genes, hence promoting transcription elongation in these AT-base enriched genomes. In *BAp*, *BSg*, *BBp* and *BCt*, DksA might also interact with the elongation factor GreA (absent in BCc). GreB (a paralogous elongation factor found in *E*. *coli*) is absent in the genomes of the five *Buchnera* strains analysed in this work. DksA and GreA show similar binding properties with the secondary channel of the RNA polymerase and show functional redundancy in *E*. *coli* but have also opposite regulatory effects for some genes [90]. GreA and DksA might have been conserved together in most *Buchera* strains for this opposite effect or also for the chaperone properties of the GreA protein [91] but this remains yet speculative. It is to note that BCc lost both DksA and GreA proteins.

Two proteins, YchA and YrbA, referred to here as “hypothetical regulators”, are conserved in the five *Buchnera* strains. YrbA is an homologous protein of BolA and was very recently renamed IbaG (induced by acid gene) in *E*. *coli*[92]. IbaG doesn’t share the morphogene properties of BolA and its enzymatic activity was not analysed but it seems to act as a transcriptional regulator to protect the cell against stress. YchA is annotated in *E*. *coli* as putative transcriptional regulators. Nothing is known about the function of these two proteins in *Buchnera*.

In the classes of “bifunctional” and “hypothetical” regulators, most of the genes (except *cspC* that is found only in *BAp*) are conserved in the five *Buchnera* strains. Expression data, published by Vinuelas *et al*. [93], revealed that *dksA*, *cspC*, *cspE* and *csrA* are all highly expressed and highly conserved in *BAp*, *ychA* is highly conserved with a low expression level, and *yrbA* is evolving quickly and is highly expressed. Since Poliakov *et al*. [66] detected the proteins DksA, CspC, CspE, YchA and YrbA by proteomics analysis of *BAp* samples, these six genes represent interesting candidates for future experimental studies.

#### Nucleoid associated proteins and topoisomerases

Seven regulators of DNA-topology are conserved in the *Buchnera* genomes whereas most of the specific transcriptional regulators have been lost. Among them, five are classified as NAPs: FIS, H-NS, HU (encoded by *hupA*), IHF (encoded by *himA* and *himD*) and YbaB, and the other two are the topoisomerases TopI and the DNA Gyrase (encoded by *gyrA* and *gyrB*).

The identity percentages between the *E*. *coli* and *Buchnera* protein sequences are high for the NAPs and their functionally important sites (when known) are all well conserved, as reported in Table 2 and illustrated in Additional file 2. One exception is the FIS protein that is composed of two domains. The N-terminal domain, required for phage integration and recombination activity, has accumulated amino acid substitutions in *Buchnera* genomes lacking recombination capability. On the other hand, the C-terminal DNA-binding domain of the protein is highly conserved (Additional file 2).

With the exception of YbaB (function unclear, as stated by Dillon and Dorman [33]), the NAPs are not equally conserved in the genomes of the five *Buchnera* strains and not all have been detected in *BAp* by proteomic analysis [66] (Table 2). We have observed that when HU is lacking as it is in *BCc*, IHF is present, and vice versa as observed in *BBp*. *BBp* shows the smallest set of NAPs (with only YbaB and HU). Hence, the conservation of a minimal set of NAPs is consistent with a pleiotropic function of the two paralogous proteins, HU and IHF, which can compensate each other in *E*. *coli*, and also with the non-viability of deletion mutants lacking HU, IHF and H-NS [94].

Analysis of the syntheny between the different *Buchnera* genomes reveals that the loss of each NAP is a specific gene deletion, as neighbouring genes on both sides are always well conserved (data not shown). Hence, these observations are consistent with the loss, in the different strains, of some selective pressure on gene compaction and global repression. Such differences of selection pressure between the *Buchnera* strains have also been reported for the transport function, and they have been correlated with the differential success of their aphid hosts [9]. *A*. *pisum* and *S*. *graminum* are modern, cosmopolitan, oligophagous and successful aphids and their *Buchnera* have retained the largest set of NAPs, whereas *B*. *pistaciae*, *C*. *cedri* and *C*. *tujafilina* are more primitive aphids, with smaller geographical distribution and slower growing rates, and their *Buchnera* have retained minimal sets of NAPs, possibly because these bacteria are thought to face less diverse physiological conditions.

NAPs are global regulators of DNA topology but they can also act as true transcription factors capable of interacting with the promoter regions of some target genes. Transcriptomic analyses revealed that mutants deleted for NAPs, or overexpressing them, modify the gene expression regulation of numerous genes (e.g., 819 and 610 for ΔFiS and ΔHNS mutants, respectively, in *E*. *coli*[19]). However, among these genes, subgroups of more specific targets are found in the RegulonDB database [47]. We have analysed, in *BAp*, the distribution of the conserved specific target genes (using *E*. *coli* as the reference ancestral model) for the five NAPs (Additional file 3). Since a neutral model of random gene deletion would predict equivalent proportions of conserved target/non-target genes for all the NAPs, no specific selection for gene conservation was found in the *Buchnera* strains analysed (Additional file 3).

*Buchnera* possess the minimal set of topoisomerases required to control chromosome supercoiling: one topoisomerase I, which removes negative supercoils without consuming ATP, and one gyrase (ATP-dependent) necessary for their introduction. In *E*. *coli*, two supplementary topoisomerases are involved in the decatenation process, allowing for chromosome separation at the end of replication. The absence of these two topoisomerases in *Buchnera* may be linked with the high ploïdy of these bacteria, as observed by Komaki and Ishikawa [95] in *BAp*. More surprising is the absence of topoisomerase I in *BBp* and *BCc*. Gil *et al*. [96] have proposed that the gyrase in *Buchnera* might have acquired a broader function and has become capable of removing or introducing negative supercoils, as suggested previously by Drolet *et al*. [97] in *E*. *coli*.

**Figure 1:**
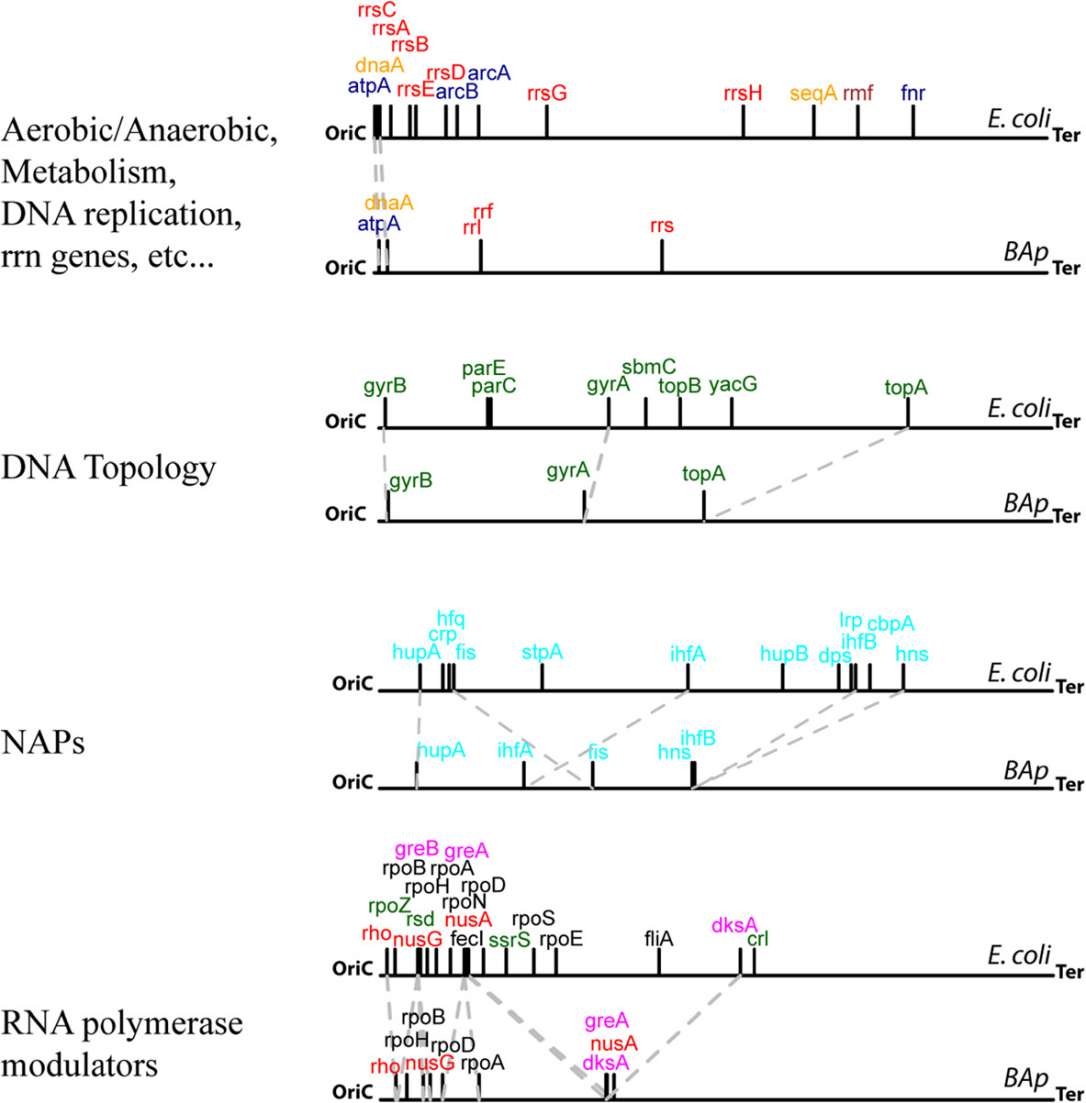
**Spatial organisation of the *BAp *chromosome compared with that of *E. coli*. **First line bars: genes involved in aerobic/anaerobic metabolism (dark blue), DNA replication (orange), rrn genes (upper red), and transition phase (brown). Second line bars: selected genes involved in the control of DNA topology (green). Third line bars: selected genes encoding NAPs (light blue). Fourth line bars: selected genes involved in modulating RNAP activity, including σ factor-utilization regulators (light green), secondary channel-binding proteins (pink), termination/elongation factors (red), and RNAP subunits (black). Grey dotted lines link pairs of orthologous genes between *BAp *and *E. coli*. Selected genes are those described by Sobetzko *et al*. [39].

Recent studies by Sobetzko *et al*. [39] show that the positioning of the different NAPs (as well as that of several other regulatory and metabolic genes) is not random on the chromosome but, instead, it is specifically associated with the DNA macrodomains that are differentially sensitive to the states of DNA-relaxation. This is observed across the range of bacterial diversity and provides an ancestral mechanistic insight into how the chromosome organisation “encodes” a spatiotemporal program to globally regulate gene expression. Although indicated by a previous genomic analysis [93], the existence of macrodomains has not been demonstrated in the *Buchnera* genomes. However, such ancestral genome organization (i.e., the relative positioning of NAPs along the chromosome) seems to be conserved in *BAp*, despite its genome erosion (Figure 1).

### Inventory of the cis-regulatory elements in *Buchnera* genomes

In *BAp*, 699 σ^70^ promoters were detected, in the 500 bp regions located upstream of the 574 ORF of the genome, which corresponds to 94% of the CDS and 96% of the TUs showing at least one significant promoter prediction. The same observations were made for *BSg*, *BBp* and *BCc* CDS where for 94%, 95% and 95% of the CDS, respectively, we were able to find σ^70^ promoters (Additional file 4). Knowing that BPROM scores are proportional to the similarity with the consensus *E*. *coli* boxes, it is worth noting that the scores predicted within the *BAp* TUs (putative internal promoters) were lower as regards the scores of the promoters located upstream of the TUs (Additional file 5A). As a comparison, in *E*. *coli*, a significant σ^70^ promoter prediction was found upstream of 89% of the CDS and 92% of the TUs, and the size distribution of the corresponding 5’ UTRs (5’ UnTranslated Region) was similar in the two organisms (186 ± 127 and 200 ± 128 bp in *BAp* and *E*. *coli* respectively), as shown in the Additional file 5B.

**Figure 2:**
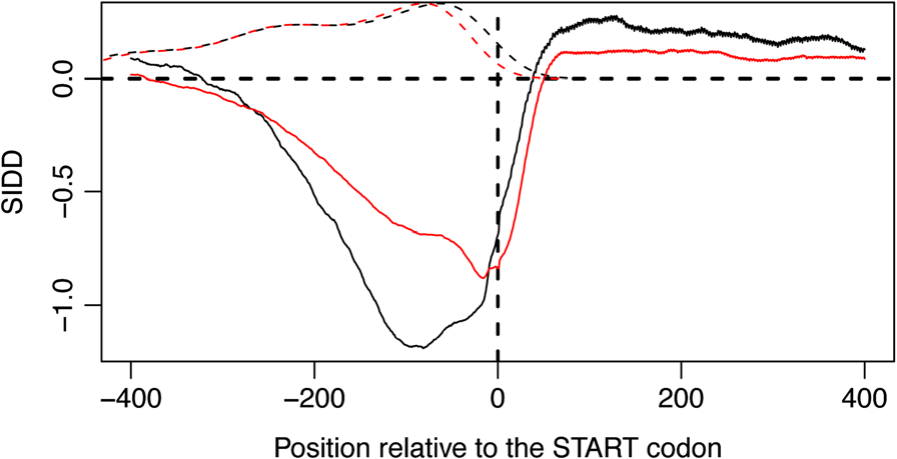
**SIDD profile of the 400 bp located around the start codon of all CDS.***BAp*: black, continuous curve; *E. coli*: red, continuous curve. The upper curves (dotted lines) depict the densities of the length of the 5’ UTR regions in *BAp *(black) and in *E. coli *(red), as determined by BPROM. No Y-coordinate scale is given for these curves: the density peaks correspond to the most frequent transcription start site positions (relative to the start codon) in the two bacteria.

In order to validate our prediction, we analyzed the SIDD profile (see below and the Methods section) of the 400 bp regions located around the start codon of all the CDS found in *BAp*, differentiating between the intra- and inter-TU regions (Figure 2). Despite the AT-richness of the intergenic regions in *BAp*, a characteristic profile with an instability sink was observed at about 100–150 bp upstream of the start codon, and this was also observed for the strains *BSg*, *BBp* and *BCc* (data not shown). Likewise, this sink is present in *E*. *coli*, where it is located around the start codon. It is important to add that internal promoters located within *BAp* TUs showed a similar profile centred around the start codon, but with higher SIDD values, i.e., they are more stable (data not shown).

Taken together, all these results (conservation of promoters, intergenic distances and thermodynamic profiles) concur with the prediction for a functional role of the σ^70^ promoters, despite the high rate of sequence evolution in *Buchnera*.

A similar analysis was performed for the σ^32^ promoters (located 500 bp upstream of CDS) and for the four *Buchnera* strains *BAp*, *BSg*, *BBp and BCc* where, respectively, 248, 238, 179 and 98 σ^32^ promoters were found. Nevertheless, it is significant that, in *BAp*, among the 45 conserved genes from the σ^32^ regulon of *E*. *coli*, only 19 (42%) had retained a recognizable σ^32^ promoter. Moreover, when comparing the putative σ^32^ regulons of these four *Buchnera* strains, they appeared to be very divergent, revealing that predicted σ^32^ promoters are not tractable in *Buchnera* without any experimental data (Additional file 6). These results are quite important as regards the possible role of σ^32^ in the AT-rich genomes of insect endosymbionts, characterised by mild transcriptional changes in response to heat shock [98,99]. It has been proposed that, in symbiotic genomes, this transcriptional regulator might control currently unknown stress signals, or it may have been transformed into an alternative vegetative σ factor in order to replace the lost σ factors (σ^24^, σ^28^, σ^38^ and σ^54^). Indeed, the very high number of σ^32^ promoters found in the *Buchnera* genomes, coupled with the inconsistency of the predicted corresponding regulons, is likely to reflect a high rate of false positives rather than the acquisition of a more generalist role for this σ factor.

A systematic search of TFBS was also performed for the three NAPs for which a known TFBS has been described (see Methods section). However, in *Buchnera*, all the predicted TFBS are under-represented as regards what would be randomly expected, so it was not possible to extract significant TFBS even for these proteins (data not shown).

### Intrinsic structural properties of the DNA molecule of *Buchnera*

**Figure 3:**
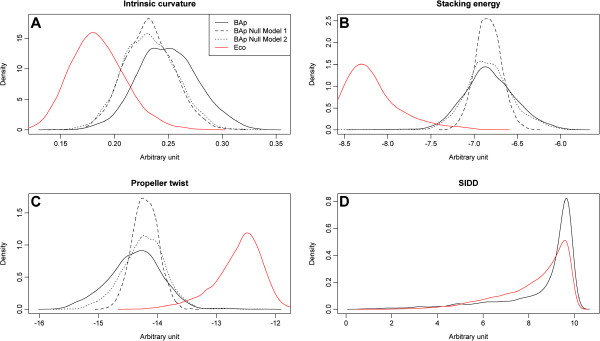
**General comparison, between *BAp *and *E. coli, *of four physical properties of the DNA molecule. A**: intrinsic curvature; **B**: base-stacking energy; **C**: base-pair propeller twist and **D**: SIDD. Global and local null models are described in the Methods section. Parameter values were estimated using 300 bp non-overlapping sliding windows.

The role of DNA negative supercoiling in the regulation of transcription activity in bacteria is now well established [19]. Here, we analysed the sequence dependent features determining some of the physical properties of the DNA molecule of *Buchnera*, namely intrinsic curvature and SIDD. These parameters enabled us to highlight the most favourable promoter regions for transcription initiation, together with base stacking energy and base-pair propeller-twisting relevant to the flexibility and stability of the DNA. Physical properties of the DNA molecule of *BAp* were compared to those of *E*. *coli* using the global and local null models of base-composition as references (see Methods section). The results are presented in Figure 3. As a direct effect of its AT-richness, the DNA molecule of *BAp* is much more curved, flexible and shows less base-pair stability compared to that of *E*. *coli*. The curvature and the flexibility provided by the propeller twist are more pronounced in *BAp* than in the global and local null models.

**Figure 4:**
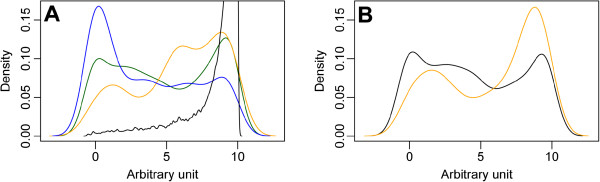
**SIDD distribution in the different intergenic regions of the *BAp *DNA molecule. **(**A**): CDS (black), divergent (blue), convergent (yellow), tandem (green). (**B**): same as A, separating the tandem region intra- transcription unit (yellow) from the inter-transcription unit regions (black).

The SIDD is less closely correlated to the AT-composition and it does not allow for discrimination between *E*. *coli* and *BAp*. In order to detect regions more prone to harbour functional promoters, we analysed the SIDD scores within the intergenic regions of *BAp*. Indeed, divergent intergenic regions (containing two 5’ UTR sequences) encode more promoters than convergent ones (containing two 3’ UTR sequences). Hence, the SIDD scores serve to separate the clearly divergent (less stable) and the convergent (more stable) intergenic regions. Interestingly, tandem regions (containing one 3’UTR and one 5’UTR), that could harbour either a TU external promoter, a TU internal promoter or even no promoter at all, reveal a bimodal distribution separating out the stable and the unstable regions (Figure 4A). We analysed, in more detail, the SIDD distribution within the tandem regions (Figure 4B) and we found that the intra-TU promoter regions were the most stable, whereas the inter-TU promoter regions still showed a bimodal distribution of stable and unstable regions. Referring to our previous work on TUs in *BAp*[26], it is probable that stable promoter regions correspond to false predicted inter-TU regions in this strain since long TUs, associating non-functionally related genes, seem to be over-abundant in *BAp*.

We analysed the correlation between the SIDD values of each gene promoter region (calculated in a window of 150 bp, located upstream of the CDS start) and the corresponding functional gene classes in *BAp*, using the *multiFun* classification [100]. Two classes were found to be significantly correlated with unstable promoter regions (Wilcox rank test): the transporter class (p-value = 0.03) and the regulator class (p-value = 2 10^-4^). Significant gene lists are given in the Additional file 7. The GC-content of these intergenic regions are not significantly different from the overall intergenic GC-content of *BAp*, hence this correlation cannot be explained only by a strand bias or a local GC-bias effect (data not shown). The association between unstable promoters and genes involved in transcription or transport was described for 38 out of 43 free living bacteria analysed by Wang and Beham [40], whereas the authors pointed out that this regulatory mechanism was lost in a group of obligate parasitic bacteria (for 14 out of the 18 analysed) including *Chlamydia* and *Mycoplasma* species.

We also looked for associations between unstable promoters and functional gene properties in a data set separating the sensitive from the insensitive genes with respect to supercoiling variations in *E*. *coli*[19,23], as well as in several expression data sets of *BAp* involving stimulations of the essential amino acid metabolism [14,15,93]. However, no significant correlation was found (data not shown) probably because sensitive genes are different between *BAp* and *E*. *coli* and because the expression data were average for the total *Buchnera* populations within aphid tissues (*i*.*e*., maternal and embryonic populations with different nutritional requirements and stress sensibility).

## Conclusions

### Towards a putative model of topological regulation of the gene expression in *Buchnera*

The inventory of the regulatory elements of *Buchnera* reveals the very low diversity of specific regulators and the conservation of several NAPs and topoisomerases. These results are also consistent with the attenuated expression profiles observed in *BAp* and *BSg* microarray experiments [12-14] since NAPs induce continuous changes in the gene transcription rates, whereas local transcription factors induce discrete changes (i.e., On/Off transcription rates) [19,101]. DNA-superhelical density has not, as yet, been measured in *Buchnera* and changes in the supercoiling induced by variations of the intracellular environment of their bacteriocytes remains speculative. Hence, the question of the functionality of DNA-topological regulators and of their links with gene expression regulation is still open.

Several evidences in favour of a selection of regulatory structures in *Buchnera* despite genome erosion are provided by this genomic analysis: (1) the conservation and the creation of new transcripton structures during genome shrinkage; (2) the conservation of a few specific transcription factors, corresponding to multifunctional enzymes, that may have acquired a broader regulatory role; (3) the conservation of the regulator DksA, in the absence of the alarmone ppGpp, possibly to enhance transcription elongation in these AT-rich genomes and perhaps to discriminate between stable and unstable promoters, as suggested by Srivatsan and Wang in *E*. *coli*[102]; (4) the conservation of several NAPs and of their positioning organisation along the chromosome; (5) the conservation of unstable promoters upstream of transport and regulatory genes.

This work has allowed us to propose some assumptions about the role of DNA topology in gene expression in *Buchnera*. Hence, AT-bias is generally considered to be either a consequence of the degeneration of the repair system in *Buchnera*[103] or for energetic selection linked to the centrality of ATP in the metabolism of the bacterium [104]. We have proposed here that the AT-richness of the DNA molecule of *Buchnera* might provide a selective advantage, giving more flexibility and curvature to the chromosome and facilitating its regulation with only a small number of global regulators, such as NAPs and topoisomerases.

Finally, the presence of unstable promoters upstream of transporter genes might allow a basal expression of the transporter genes in *BAp*, even when conditions are unfavourable (potentially for importing metabolic precursors), and an over-expression of these genes when the energetic level of the cell is high (potentially to export metabolites for the host). Indeed, the ATP/ADP ratio, partly controlling the gyrase activity, is linked to the superhelicity of the DNA molecule in that when the ratio is high the level of DNA-supercoiling is high and DNA strands are destabilized by the constraints increasing the activity of most promoters. On the contrary, when the ratio is low the DNA molecule is more relaxed and only unstable promoters would be active.

Experimental studies will be needed to test whether such ancestral gene expression regulation by DNA-topology remains functional in *BAp* and in other *Buchnera* with even smaller genomes and could be a preponderant mechanism of gene transcription regulation in these bacterial cells with a very small diversity of transcription factors. Hence, DNA-topology could regulate some important symbiotic functions, as well as whether superhelical changes may have occurred during the few contrasted physiological states imposed on *Buchnera* by their integration into the aphid life cycle (e.g., the establishment of symbiosis during embryonic development, the growing phase and activation of symbiotic metabolism, decaying symbiosis in old aphids, etc.).

## Abbreviations

*BAp*, *BBp*, *BCc*, *BCt*, *BSg*: *Buchnera aphidicola* from the species *Acyrthosiphon pisum*, *Baizongia pistacea*, *Cinara cedri*, *Cinara tujafilina* and *Schizaphis graminum*; CDS: Coding DNA Sequence; GO: Gene ontology; HTH: Helix-turn-helix; NAP: Nucleoid associated protein; SIDD: Stress-induced duplex destabilization; TFBS: Transcription factor binding site; TU: Transcription unit; UTR: Untranslated region.

## Competing interests

The authors declare that they have no competing interests.

## Authors’ contributions

LB, FC and HC conceived and designed the study. LB performed the analyses. LB, FC and HC analyzed the data, contributed to the text, Tables and Figures, and wrote the paper. All authors read and approved the final version of the manuscript.

## Supplementary Material

Additional file 1 **(Figure): Functional analysis (GO terms) of *BAp *genes.** Click here for file

Additional file 2 **(Figure): Conservation of the protein sequences of the six NAP encoding genes found in *Buchnera*.** Click here for file

Additional file 3 **(Table): Number (#) of specific NAP target genes in *E*. *coli *(as found in the RegulonDB) and of the orthologous genes (putative conserved targets) in the four *Buchnera *strains *BAp*, *BSg*, *BBp *and *BCc*.** Click here for file

Additional file 4 **(Table): Number (#) of predicted σ**^70 ^**promoters in the four strains of *Buchnera*.** Click here for file

Additional file 5 **(Figure): Distribution of σ**^70 ^**prediction scores and 5’UTR lengths in *BAp *and *E*. *coli*.** Click here for file

Additional file 6 **(Figure): Venn-diagram of the putative σ**^32 ^**regulons in *Buchnera*.** Click here for file

Additional file 7 **(Table): Transporter (T) and regulatory (R) genes of *BAp *significantly correlated with low SIDD values (i.e., associated with unstable promoter regions).** Click here for file
