# Supplementary material for: Genomic analysis of the regulatory elements and links with intrinsic DNA structural properties in the shrunken genome of Buchnera
Source: BMC Genomics. 2013 Feb 1;14:73. doi: 10.1186/1471-2164-14-73 (PMC3571970; doi:10.1186/1471-2164-14-73)
Supplement: Additional file 1 — (Figure): Functional analysis (GO terms) of BAp genes. [file 1471-2164-14-73-S1.pdf]

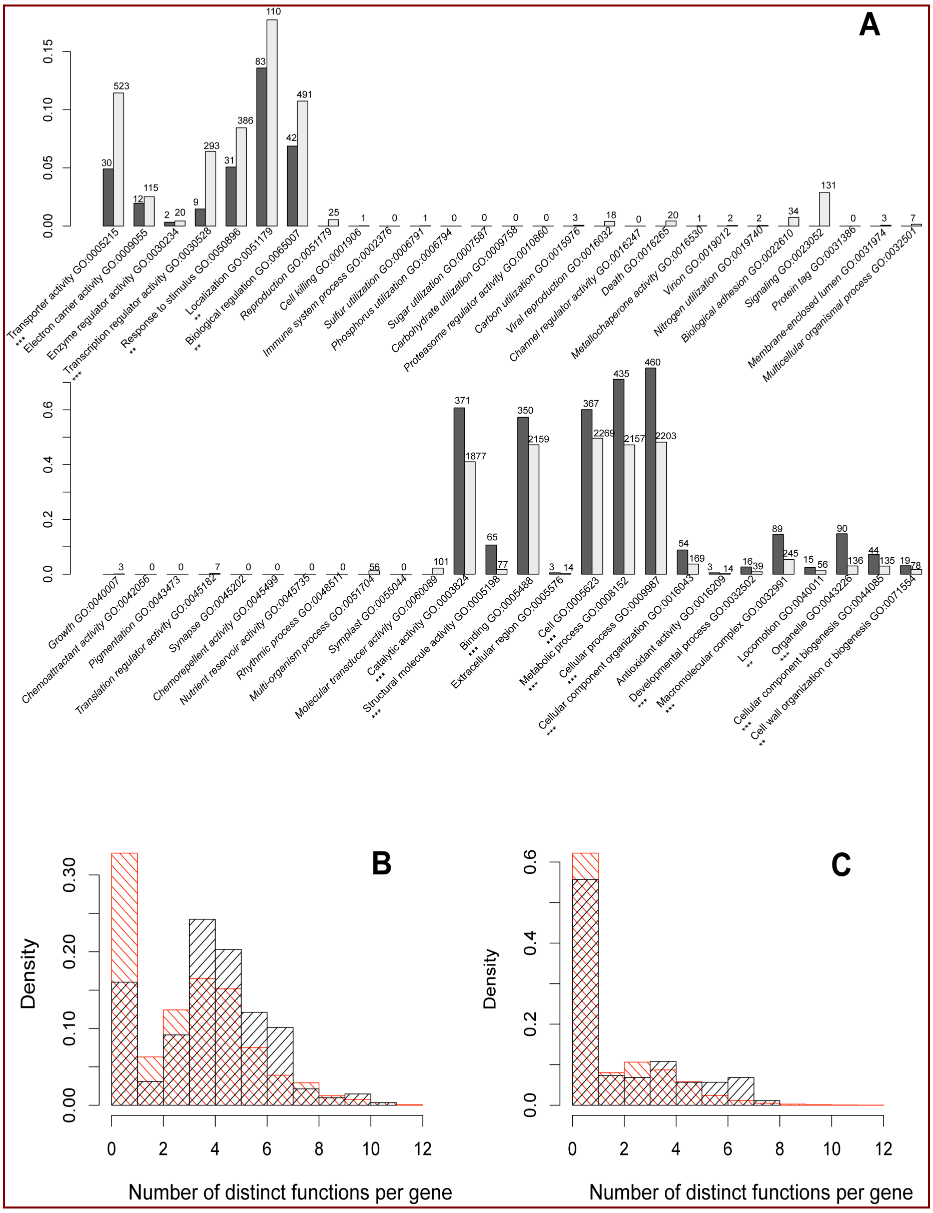

Additional file 1. Functional analysis (Gene Ontology terms) of *BAp* genes. (A) Comparison of gene proportions allocated to Gene Ontology functional classes at level 3 between *E. coli* (white rectangles) and *Buchnera* (black rectangles). Significant differences (chi-square proportion tests) were annotated as follows: * pvalue < 0.01, ** pvalue < 0.001 and *** pvalue < 0.0001. (B) Distribution of the number of GO terms (level 3) associated with genes in *Buchnera* (black) and *E. coli* (red), distributions are significantly different (Wilcoxon-test pvalue < $2\ 10^{-16}$). (C) Same as B after removal of the metabolism GO term GO:0008152, the distributions are similar (Wilcoxon distribution test pvalue = 0.07).
