# Supplementary material for: Genomic analysis of the regulatory elements and links with intrinsic DNA structural properties in the shrunken genome of Buchnera
Source: BMC Genomics. 2013 Feb 1;14:73. doi: 10.1186/1471-2164-14-73 (PMC3571970; doi:10.1186/1471-2164-14-73)
Supplement: Additional file 2 — (Figure): Conservation of the protein sequences of the six NAP encoding genes found in Buchnera. [file 1471-2164-14-73-S2.pdf]

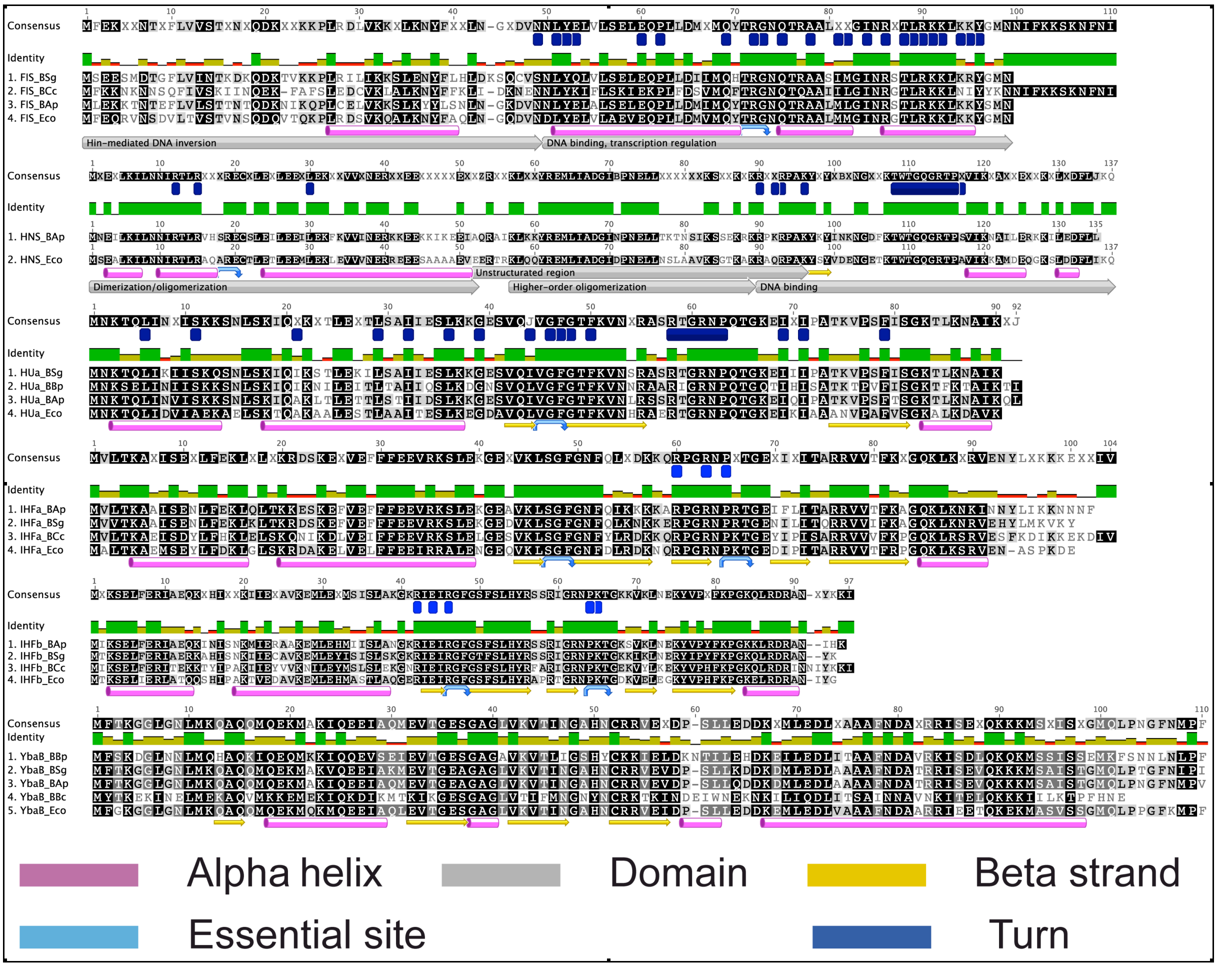

Additional file 2. Conservation of the protein sequences of the six NAPs encoding genes found in *Buchnera*. Blue squares below the consensus sequence represent functionally important amino acids. Sequence similarity is presented following a gradient color from red (low similarity) to green (identity). Colour tubes below the sequence alignment represent the different protein domains: alpha helix (pink), specific active domain (grey), beta strand (yellow), essential site (light blue), turn (dark blue).
