# Supplementary material for: Genomic analysis of the regulatory elements and links with intrinsic DNA structural properties in the shrunken genome of Buchnera
Source: BMC Genomics. 2013 Feb 1;14:73. doi: 10.1186/1471-2164-14-73 (PMC3571970; doi:10.1186/1471-2164-14-73)
Supplement: Additional file 3 — (Table): Number (#) of specific NAP target genes in E. coli (as found in the RegulonDB) and of the orthologous genes (putative conserved targets) in the four Buchnera strains BAp, BSg, BBp and BCc. [file 1471-2164-14-73-S3.pdf]

Additional file 3. Number (#) of specific NAPs target genes in *E. coli* (as found in the RegulonDB) and of the orthologous genes (putative conserved targets) in the four *Buchnera* strains *BAp*, *BSg*, *BBp* and *BCc*.

	# of *E. coli* target genes	# of conserved targets in *BAp*	# of conserved targets in *BSg*	# of conserved targets in *BBp*	# of conserved targets in *BCc*
**Fis**	215 (0.041 %)[a]	57 (0.093 %)	56 (0.096 %)	51 (0.094 %)	27 (0.068 %)
**H-NS**	145 (0.027 %)	10 (0.016 %)	9 (0.015 %)	8 (0.015 %)	3 (0.008 %)
**HU**	9 (0.002 %)	0 (0%)	0 (0%)	0 (0%)	0 (0%)
**IHF**	216 (0.041 %)	34 (0.056 %)	34 (0.058 %)	25 (0.046 %)	26 (0.065 %)
Chi^2 tests[b]	-	NS	NS	NS	NS

[a] percentages are estimated on the total number of genes for each organism (5305 for *E. coli*, 610 for *BAp*, 582 for *BSg*, 541 for *BBp* and 397 for *BCc*). [b] Chi-square tests comparing the four *Buchnera* strains with *E. coli*. NS: not significant (pvalue > 0.05). YbaB was not included in the analysis, as no target is known for this protein.
