# Supplementary material for: Genomic analysis of the regulatory elements and links with intrinsic DNA structural properties in the shrunken genome of Buchnera
Source: BMC Genomics. 2013 Feb 1;14:73. doi: 10.1186/1471-2164-14-73 (PMC3571970; doi:10.1186/1471-2164-14-73)
Supplement: Additional file 4 — (Table): Number (#) of predicted σ70 promoters in the four strains of Buchnera. [file 1471-2164-14-73-S4.pdf]

Additional file 4. Number (#) of predicted σ^{70} promoters in the four strains of *Buchnera*.

***Buchnera* strain**	**# of predicted σ^{70} promoters**	**# of genes with at least one σ^{70} promoter**	**# of genes with σ^{70} and σ^{32} promoters**	**# of genes having σ^{70} and σ^{32} promoters, the σ^{70} promoter being closer to the start codon**
BAp	699	574 (94%)	231	163
BSg	691	583 (94%)	231	144
BBp	621	520 (95%)	179	97
BCc	452	376 (95%)	91	56
