# Supplementary material for: Genomic analysis of the regulatory elements and links with intrinsic DNA structural properties in the shrunken genome of Buchnera
Source: BMC Genomics. 2013 Feb 1;14:73. doi: 10.1186/1471-2164-14-73 (PMC3571970; doi:10.1186/1471-2164-14-73)
Supplement: Additional file 5 — (Figure): Distribution of σ70 prediction scores and 5’UTR lengths in BAp and E. coli. [file 1471-2164-14-73-S5.pdf]

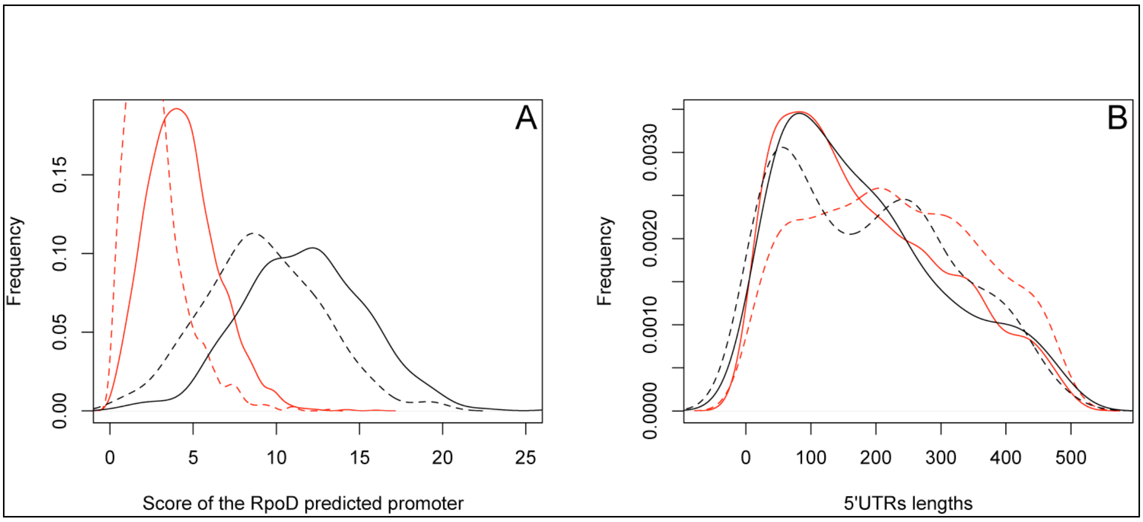

Additional file 5. Distribution of σ^{70} prediction scores and 5'UTR lengths in *BAp* and *E. coli*. (A) Distribution of σ^{70} prediction scores in *BAp* (black) and *E. coli* (red) at the beginning of transcription units (continuous lines) and within transcription units (dotted lines); (B) Distribution of the size of the predicted 5'UTR. Scores and UTR lengths are calculated by BPROM.
