# Supplementary material for: Genomic analysis of the regulatory elements and links with intrinsic DNA structural properties in the shrunken genome of Buchnera
Source: BMC Genomics. 2013 Feb 1;14:73. doi: 10.1186/1471-2164-14-73 (PMC3571970; doi:10.1186/1471-2164-14-73)
Supplement: Additional file 6 — (Figure): Venn-diagram of the putative σ32 regulons in Buchnera. [file 1471-2164-14-73-S6.pdf]

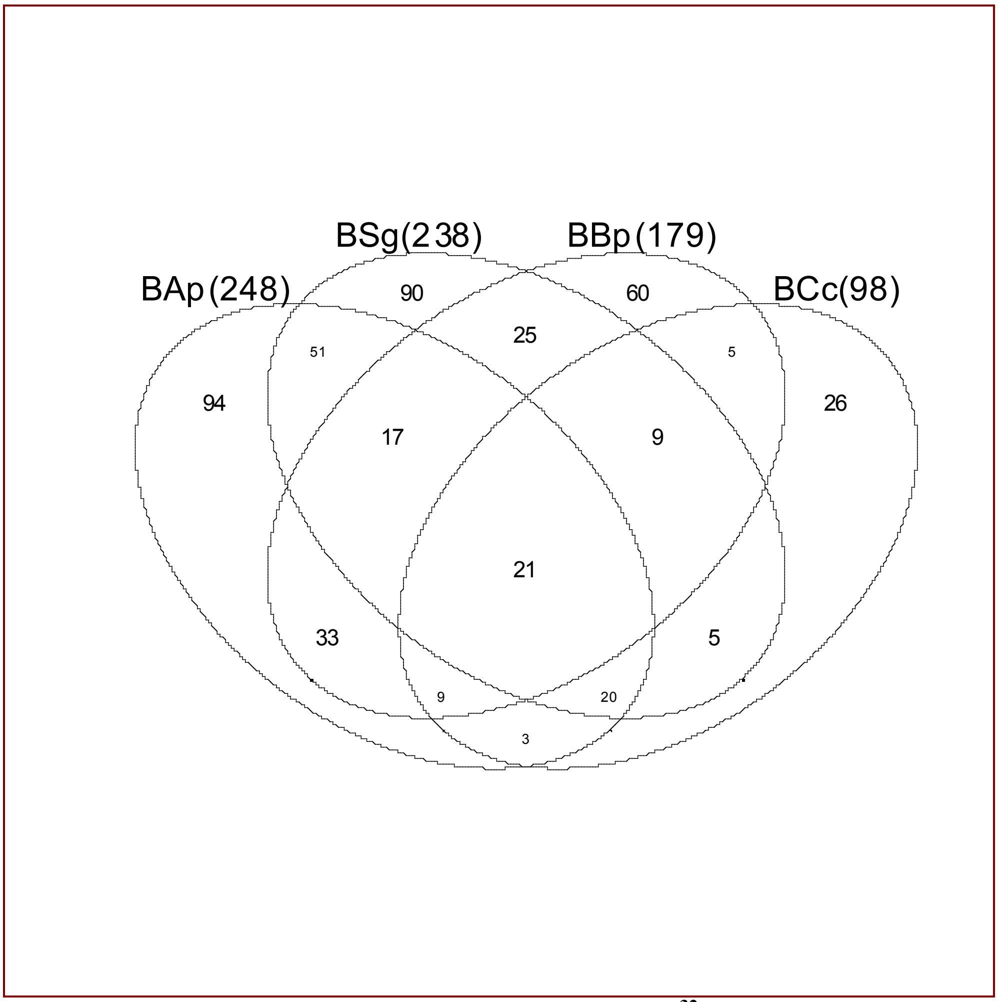

Additional file 6. Venn-diagram of the putative σ^{32} regulons in *Buchnera*. Sets of genes with a predicted σ^{32} promoter in the 500 bp located upstream of the start codon in the four *Buchnera* strains *BAp*, *BSg*, *BBp* and *BCc*. The total number of predictions for each strain is given next to the strain names.
