# Supplementary material for: Genomic analysis of the regulatory elements and links with intrinsic DNA structural properties in the shrunken genome of Buchnera
Source: BMC Genomics. 2013 Feb 1;14:73. doi: 10.1186/1471-2164-14-73 (PMC3571970; doi:10.1186/1471-2164-14-73)
Supplement: Additional file 7 — (Table): Transporter (T) and regulatory (R) genes of BAp significantly correlated with low SIDD values (i.e., associated with unstable promoter regions). [file 1471-2164-14-73-S7.pdf]

Additional file 7. Transporter (T) and regulatory (R) genes of *BAp* significantly correlated with low SIDD values of (i.e., associated with unstable promoter regions).

Gene Name	Gene Class	SIDD	Gene Name	Gene Class	SIDD
atpC	T	7.973626	*znuC*	T	1.852131
dnaA	R	1.983502	*htpX*	T	2.043931
yidC	T	2.183305	*cspC*	R	5.848372
groES	T	-0.624856	*yoaE*	T	1.045343
mopA	T	9.335936	*ompA*	T	-0.106481
rpoH	R	-0.111287	*mviN*	T	-0.165919
hupA	R	-0.410921	*flgN*	T	4.663798
secE	T	1.420129	*flgA*	T	3.194396
secB	T	2.587975	*flgJ*	T	5.821109
rpoD	R	-0.292089	*flgK*	T	-0.40305
crr	T	8.800626	*ptsG*	T	7.364209
ptsI	T	9.276389	*ompF*	T	0.750697
ptsH	T	1.614172	*uup*	T	8.572132
fliE	T	-0.622341	*pepA*	R	-0.767923
fliM	T	4.798038	*secG*	T	-0.49429
fliR	T	8.752966	*yrbA*	R	1.463955
ydiK	T	-0.756372	*ffh*	T	1.98595
himA	R	6.928056	*fis*	R	0.707795
yajC	T	8.343122	*alaS*	R	4.002205
yabI	T	1.583825	*csrA*	R	3.583716
lspA	T	5.821346	*ygfZ*	R	5.984634
dnaK	T	8.710727	*ybeX*	T	5.986981
ychA	R	-0.574629	*yhgN*	T	-0.17535
dksA	R	-0.534075	*mscS*	T	-0.11272
secA	T	4.372674	*yajR*	T	2.690397
flhB	T	-0.60933	*bolA*	R	-0.709555
flhA	T	9.526875	*tig*	T	0.228236
lepB	T	9.358191	*mdl*	T	1.041389
lepA	T	-0.036418	*mdlB*	T	9.190276
ycfC	R	8.365733	*cspE*	R	-0.118034
ychE	T	2.783647	*secY*	T	5.376038
hns	R	0.229495	*tsgA*	T	-0.011042
yciC	T	2.159532	*yhgI*	T	4.782152
yedA	T	-0.287544	*mtlA*	T	1.491904
lolC	T	5.660755	*pitA*	T	1.845044
lolD	T	9.431697	*ynfM*	T	6.035342
glpF	T	8.041876	*yfgM*	T	3.384412
himD	R	-0.192268			
